# Cytopathic Effects Incited by Viroid RNAs and Putative Underlying Mechanisms

**DOI:** 10.3389/fpls.2012.00288

**Published:** 2013-01-07

**Authors:** Francesco Di Serio, Angelo De Stradis, Sonia Delgado, Ricardo Flores, Beatriz Navarro

**Affiliations:** ^1^Istituto di Virologia Vegetale, UOS Bari, Consiglio Nazionale delle RicercheBari, Italy; ^2^Instituto de Biología Molecular y Celular de Plantas, Universidad Politécnica de Valencia-Consejo Superior de Investigaciones CientíficasValencia, Spain

**Keywords:** cell wall, chloroplast, non-coding RNAs, pathogenesis, plasmalemmasome, RNA silencing

## Abstract

Viroids are infectious agents identified only in plants so far. In contrast to viruses, the genome of viroids is composed of a tiny circular RNA (250–400 nt) not coding for proteins, but containing in its compact structure all the information needed for parasitizing the transcriptional and RNA trafficking machineries of their hosts. Viroid infections are frequently accompanied by cellular and developmental disorders that ultimately result in macroscopic symptoms. The molecular events linking the structural domains of viroid RNAs with cellular and macroscopic alterations remain largely unexplored, although significant progress has been lately achieved in one specific viroid-host combination, highlighting the ability of viroids to strongly interfere with their host RNA regulatory networks. Cytopathic effects induced by nuclear-replicating viroids, which were investigated since early studies on viroids, consist in irregular proliferations of cell membranes (paramural bodies or plasmalemmasomes), cell wall distortions, and chloroplast malformations. Different alternatives have been proposed regarding how these cytological alterations may influence the onset of macroscopic symptoms. Recently, the cytopathology and histopathology incited by a chloroplast-replicating viroid have been investigated in depth, with defects in chloroplast development having been related to specific molecular events that involve RNA silencing and impairment of chloroplast ribosomal RNA maturation. On this basis, a tentative model connecting specific cytopathologic alterations with symptoms has been put forward. Here, early and more recent studies addressing this issue will be reviewed and reassessed in the light of recent advances in the regulatory roles of small RNAs.

## Introduction

Viroids are infectious agents composed exclusively of a small (246–401 nt), circular, and highly structured RNA able to replicate autonomously and move systemically in their host plants, wherein they frequently elicit macroscopic symptoms (Flores et al., 2005; Ding, 2009). The approximately 30 viroid species reported so far have been assigned to the taxonomic families *Pospiviroidae*, grouping the type species *Potato spindle tuber viroid* (PSTVd) and many others that replicate and accumulate in the nucleus, and *Avsunviroidae*, clustering the type species *Avocado sunblotch viroid* (ASBVd) and three other viroids replicating and accumulating in plastids (mainly chloroplasts; Owens et al., 2011). Members of both families induce severe diseases in certain hosts, although in some viroid-host combinations the infection remains latent (Flores et al., 2011). Viroid diseases are characterized by macroscopic symptoms that may include stunting, alterations affecting leaves (epinasty, vein clearing, distortion, discoloration, mottling, and necrosis) and bark (cankers, scaling, cracking), and malformations of tubers, flowers, and fruits (in the last two organs frequently accompanied with color breaking). In rare cases viroids may cause the death of the plant.

In contrast to viruses, all the available data support that viroids do not code for any protein and, therefore, their replication, trafficking, and pathogenesis rely on the interplay between the invading RNA and host factors. There is evidence for members of both families supporting the direct interaction of viroid RNAs with host proteins (Daròs and Flores, 2002; Martínez de Alba et al., 2003; see for a review Owens and Hammond, 2009). These interactions are most likely mediated by structural domains that, mimicking those contained in cellular RNAs, allow viroid RNAs to usurp and redirect enzymes and other host components to their replication and trafficking. Structural elements in the genomic viroid RNA and their proposed role(s) in conferring functional properties to these infectious agents have been recently reviewed (Flores et al., 2012). Some of these elements have been shown to regulate specifically cell-to-cell (Qi et al., 2004; Takeda et al., 2011) and long distance (Zhong et al., 2007, 2008) movement of PSTVd through the plasmodesmata and phloem, respectively, while others have been implicated in replication or in pathogenesis (see for a review Navarro et al., 2012a).

Whether the macroscopic symptoms result from interaction(s) between the genomic viroid RNA and host factor(s) detracted from their physiological functions is not known. Alternatively, post-translational triggering of signaling cascades by RNA-activated phosphorylation of host proteins has been proposed as the primary event of viroid pathogenesis (Hiddinga et al., 1988; see for a review Owens and Hammond, 2009). In addition to many studies supporting the ability of viroids to modify host gene expression during infection (Conejero et al., 1990; Itaya et al., 2002; Tessitori et al., 2007; Wang et al., 2011; Owens et al., 2012; Rizza et al., 2012; see for a review Owens and Hammond, 2009), other hypotheses based on the interference of viroid RNAs with the plant RNA silencing machinery have been proposed for their pathogenesis in the last few years (see below). Models on how viroids may elicit macroscopic symptoms have been described in previous reviews (Flores et al., 2005; Ding, 2009; Owens and Hammond, 2009; Navarro et al., 2012a). Here, we will address viroid pathogenesis from a different perspective, focusing on the cytological alterations induced by viroids in infected cells and taking into account recent advances in the plant-viroid interplay achieved by genome-wide technologies.

## Viroid Infection is Frequently Accompanied by Cytological Alterations

**Figure 1:**
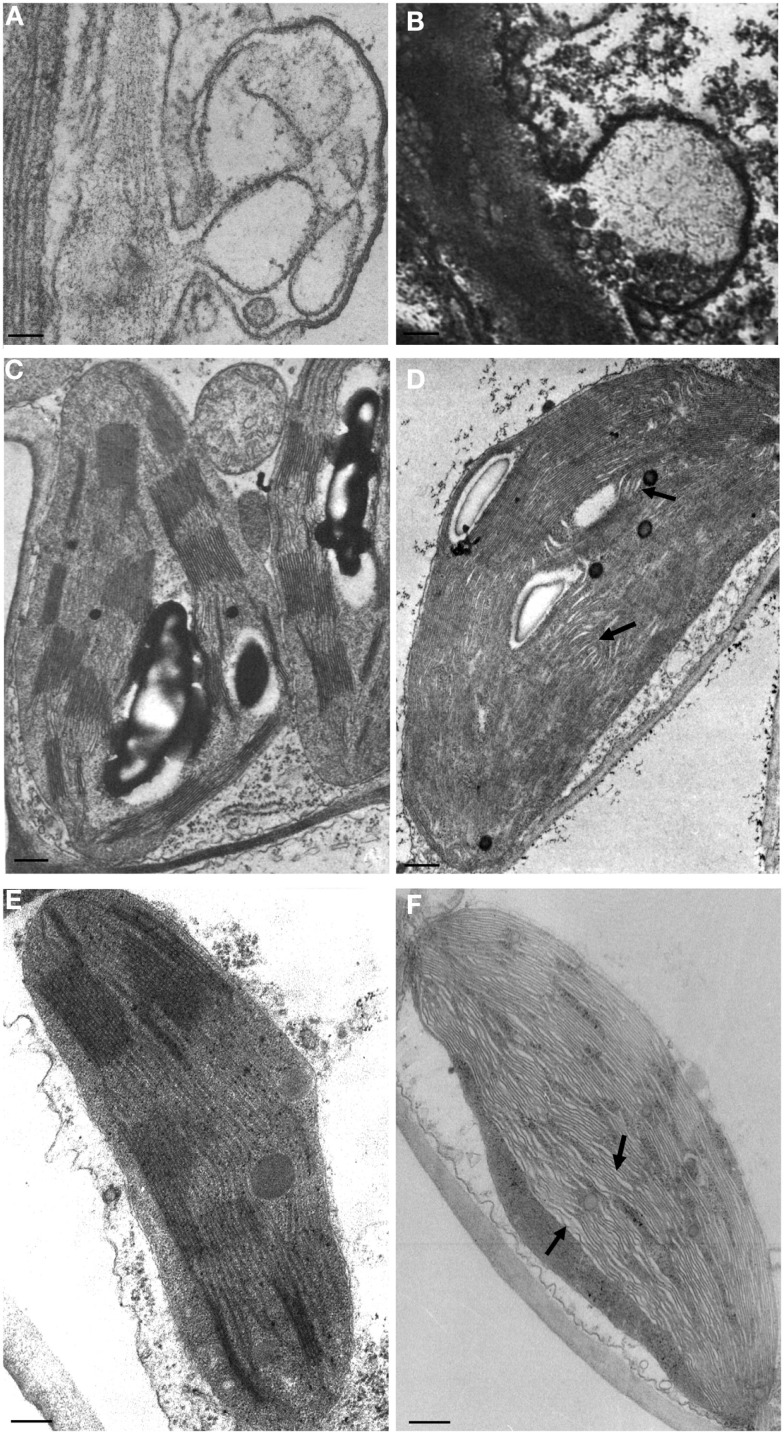
**Cytopathic effects associated to viroid infection**. Plasmalemmasomes in tomato infected by PSTVd **(A)**, and in avocado infected by ASBVd **(B)**. Normal and malformed chloroplasts with irregularly stacked thylakoids and wide interspaces (denoted with arrows) in healthy and PSTVd-infected tomato [**(C,D)** respectively], and in peach healthy and infected with a PLMVd latent variant [**(E,F)** respectively). Bar = 50 nm. **(A,C,D)** have been reproduced (with modifications) from Hari (1980; Copyright The American Phytopathological Society), and **(B)** from Da Graça and Martin, 1981; Copyright John Wiley and Sons), in all cases with permission.

Cytopathic effects of viroid infections were first reported by Semancik and Vanderwoude (1976) in the experimental host *Gynura aurantiaca* infected by *Citrus exocortis viroid* (CEVd), a member of family *Pospiviroidae*. Using electron microscopy, these authors identified in symptomatic leaves paramural bodies with electron density similar to plasma membranes that they termed plasmalemmasomes (PSs), which later on were also observed in tomato infected by PSTVd (Hari, 1980; Figure 1A). These invaginations of the plasmalemma into the cytoplasm were rare in asymptomatic leaves of infected plants, and absent in leaves that had reached maturity before viroid infection and in uninfected controls. Therefore, PSs were considered the primary cytopathic effect of CEVd infection (Semancik and Vanderwoude, 1976). They were found in all cell types, including mesophyll, epidermis, companion cells, and phloem and xylem parenchyma of vascular bundles, and were closely associated with the leaf epinasty and blistering caused by the viroid. The morphology of PSs was largely variable, with those from mature cells being prevalently located at the cell wall-plasma membrane interface and displaying multiple membrane layers. In contrast, PSs from immature cells were of smaller size and contained roundish granules. In some instances, PSs were also observed in contact with plasmodesmata, occasionally in opposite positions separated by the cell wall (Semancik and Vanderwoude, 1976).

A few years later, these data were questioned because PSs were recognized in both symptomatic and uninfected *G. aurantiaca* plants (Wahn et al., 1980). In particular, PSs were detected with the same frequency in CEVd-infected and healthy controls. Moreover, a distinction was made between vesicular and tubular PSs depending on their ultrastructure. Formation of PSs as the primary cytopathic effect induced by CEVd in *G. aurantiaca* was excluded in this study, but the shape and internal structure of both vesicular and tubular PSs were different in CEVd-infected and healthy leaf tissues (Wahn et al., 1980). In particular, PSs from diseased tissues contained vesicles strikingly irregular in their shape, size, and number, while tubular PSs contained malformed tubules; similar alterations were never observed in healthy controls and therefore appeared viroid-induced. Moreover, another interesting cytopathic effect of CEVd infection in *G. aurantiaca* was identified in this same study: the conspicuous distortion and irregular thickness of cell walls, which produced irregular cell shapes in symptomatic tissues (Wahn et al., 1980; see also below). Intriguingly, malformed PSs were only found in cells with altered walls, suggesting a correlation between these two subcellular alterations.

The role of PSs in viroid pathogenesis remains controversial. As indicated above, paramural bodies were subsequently reported as a cytopathic effect of PSTVd infection in tomato leaves (Figure 1A; Hari, 1980), thus extending their presence to viroid-host combinations other than CEVd and *G. aurantiaca*. PSs were later observed also in chlorotic tissues of chrysanthemum infected by *Chrysanthemum stunt viroid* (CSVd), another member of the family *Pospiviroidae* (Rosenberg de Gómez et al., 1985). On the other hand, Marton et al. (1982) observed in CEVd-infected tomato callus cells a significantly higher frequency of PSs that was not regarded as viroid-specific but rather a secondary (and non-specific) effect of viroid infection. Similarly, the higher number of PSs detected by Gruner and Santore (1991) in PSTVd-infected tomato leaves with respect to healthy controls was considered as a non-specific physiological response to diverse abiotic and biotic stresses. Interestingly, an increase in the number and size of paramural bodies with respect to healthy controls was also reported in ASBVd-infected avocado tissues (Figure 1B; Da Graça and Martin, 1981), thus showing that alterations in PSs are not restricted to infections by nuclear-replicating viroids.

The discrepancies regarding PSs do not apply to the cell wall alterations first reported in *G. aurantiaca* infected by CEVd (Wahn et al., 1980). Similar distorted, undulated cell walls with variable thickness were also observed in tomato infected by CEVd (Marton et al., 1982), chrysanthemum infected by CSVd (Rosenberg de Gómez et al., 1985), and hop and cucumber infected by *Hop stunt viroid* (HSVd; Momma and Takahashi, 1982), indicating that this is the most common cytopathic effect induced by several members of the family *Pospiviroidae*. In hop plants, these structural alterations were absent in shoot tips (0.2 mm long, consisting of the apical meristem and two pairs of primordial leaves), but present starting from the third leaf primordium, showing that they appear at early leaf developmental stages (Momma and Takahashi, 1983). Anomalies in cell wall structures are also consistent with: (i) modifications in wall composition of CEVd-infected tomato cells with respect to their healthy counterparts (Wang et al., 1986), and (ii) a marked reduction in the number of protoplasts, released from CEVd-containing tomato tissues and non-differentiated calli treated with enzymes for disrupting cell walls, with respect to healthy controls (Marton et al., 1982). However, no clear differences between the cell walls of healthy and PSTVd-infected tomato leaves were observed by Gruner and Santore (1991).

Early studies on viroid cytopathology also showed structural defects in chloroplasts induced by nuclear-replicating viroids, including PSTVd (Hari, 1980), CSVd (Rosenberg de Gómez et al., 1985), and HSVd (Momma and Takahashi, 1982). These findings suggest that viroids with preferential accumulation in one specific organelle can interfere with the development and presumably the function of other cell organella. When compared to healthy controls (Figure 1C), abnormal development and organization of the chloroplast membranes, with distorted and irregularly stacked thylakoids, were observed in PSTVd-infected cells (Figure 1D). Interestingly, similar alterations have been also reported in the chloroplasts from asymptomatic tissues infected by a chloroplast-replicating viroid, *Peach latent mosaic viroid* (PLMVd; Figure 1F), but not in the healthy controls (Figure 1E; Rodio et al., 2007). Whether this structural defect is induced specifically by viroid infection or is a more general plant response to biotic stresses remains an open question.

## Linking Viroid-Induced Cytopathic Effects with Macroscopic Symptoms and the Underlying Biochemical Pathways

**Figure 2:**
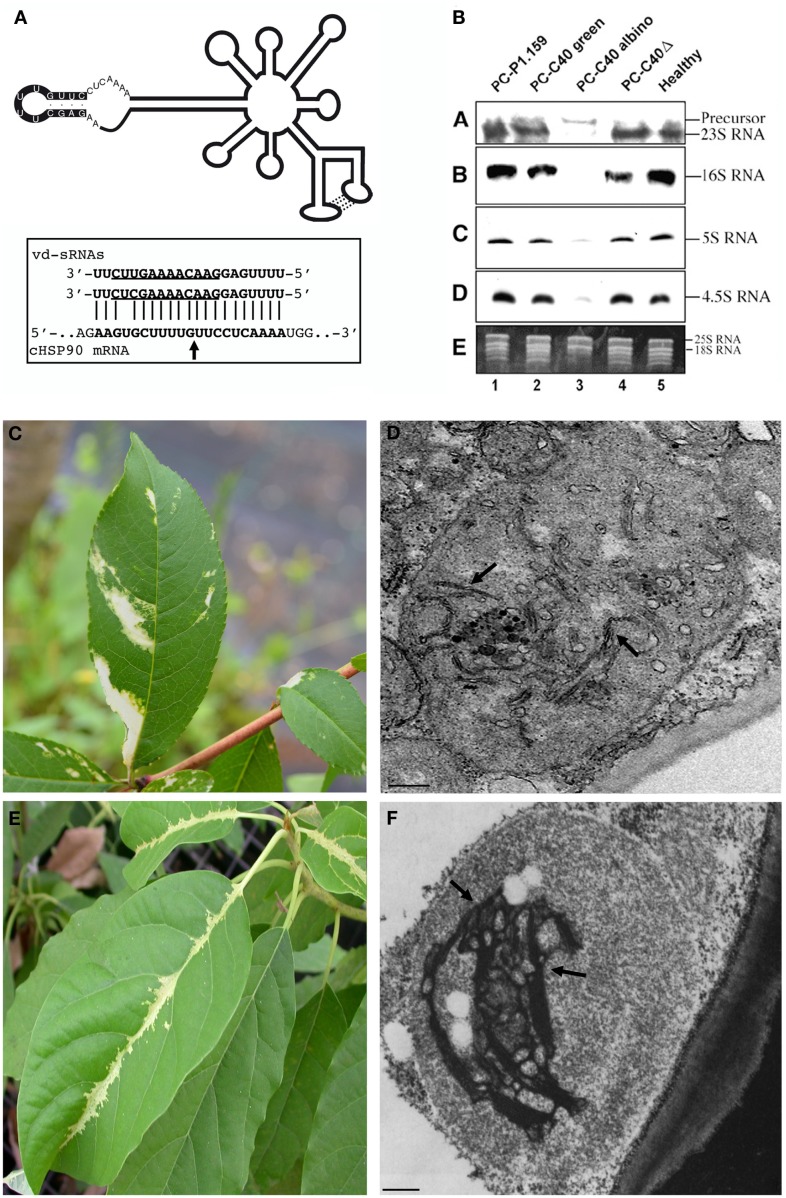
**Association of cytopathic effects and biochemical lesions with macroscopic symptoms in viroid infections**. **(A)** Schematic representation of the secondary structure predicted for a PLMVd variant inducing PC, with the nucleotides forming the PC-associated insertion denoted by a black background. (Inset) Two PLMVd (-) sRNAs mapping at this insertion target for cleavage the mRNA coding for the chloroplastic heat-shock protein 90 (cHSP90) as predicted by RNA silencing; arrow marks the predicted and validated cleavage site (Navarro et al., 2012b). **(B)** Accumulation of plastid rRNAs in GF-305 peach leaves infected by latent (PC-P1.149 and PC-C40Δ) and PC-inducing (PC-C40) variants as revealed by Northern-blot hybridizations with cDNA probes specific for 23, 16, 5, and 4.5S rRNAs; the impaired accumulation and processing of plastid rRNAs is associated with the albino phenotype (Rodio et al., 2007). **(C)** Severe chlorosis induced by a PC-inducing variant of PLMVd and **(D)** altered plastid with rudimentary thylakoid membranes (arrows) observed in chlorotic areas. **(E)** Severe chlorosis induced by ASBVd and **(F)** altered plastid with rudimentary thylakoid structures (arrows), slightly more organized than in PC, in the corresponding bleached areas. Bar = 50 nm. **(A,B,F)** have been reproduced with permissions from Navarro et al., 2012b; Copyright John Wiley and Sons), Rodio et al., 2007; Copyright American Society of Plant Biologists, www.plantcell.org) and Da Graça and Martin (1981), respectively, in all cases with permission.

Among nuclear-replicating viroids, a correlation between subcellular alterations and macroscopic symptoms was proposed for certain viroid-infected hosts, such as *G. aurantiaca*, hop, and cucumber, in which cell wall defects were accompanied by leaf distortions (Wahn et al., 1980; Momma and Takahashi, 1982). However, the most conspicuous evidence that cytopathic effects may have a direct link with macroscopic symptoms has been obtained for the chloroplast-replicating PLMVd (for a review see Flores et al., 2006). Some variants of this viroid, which contain a specific pathogenic determinant consisting of an inserted hairpin of 12 nt (Figure 2A), cause peach calico (PC), a severe disease characterized by an extreme chlorosis (albinism) of leaves (Figure 2C), stems, and fruits (Malfitano et al., 2003; Rodio et al., 2006). In albino leaf tissues from PLMVd-infected peach trees, altered plastids with irregular shape and size and with rudimentary thylakoids, thus resembling proplastids, were observed by electron microscopy (Figure 2D; Rodio et al., 2007). Further analyses revealed that structural alterations, namely rudimentary thylakoids and presence of vesicles, are already evident in most proplastids from meristematic cells of the albino shoot apices, supporting the notion that an early step of chloroplast development is specifically impaired by PLMVd variants inducing PC. These alterations have never been found in green leaf tissue and shoot apices of plants infected by PC-inducing variants or by other mosaic-inducing or latent variants, thus confirming their close association with the albino phenotype (Rodio et al., 2007). In addition, these structural alterations have been closely associated with impaired processing and accumulation of plastid rRNAs in the albino tissues (Figure 2B), a molecular defect also reported in variegated mutants of some plants (Rodio et al., 2007). Therefore, a macroscopic symptom, the corresponding cytological defects, and the possible biochemical pathway, have been closely related to the presence of a specific structural domain (the inserted hairpin) in the infecting viroid RNA.

Interestingly, another chloroplast-replicating viroid, ASBVd, may induce a severe chlorosis (bleaching) in its natural host avocado. This symptomatology, which resembles closely PC (Figure 2E), has been associated with plastid ultrastructural defects partially similar to those reported in PC, but with rudimentary thylakoid membranes slightly more organized (Da Graça and Martin, 1981; Figure 2F). Moreover, ASBVd sequence variants from bleached tissues are slightly different from those accumulating in non-symptomatic tissues (Semancik and Szychowski, 1994) and, based on our preliminary results, accumulation of plastid rRNA appears also impaired in bleached tissues (Di Serio and Flores, unpublished data). Altogether, these data establish a particularly intriguing parallelism between PLMVd and ASBVd pathogenesis.

## Cytopathic Effects are Associated with Modifications in Gene Expression

The triggering events and molecular mechanisms underlying viroid pathogenesis are still largely unknown, but it is generally accepted that these RNAs induce modifications of host gene expression that, ultimately, lead to macroscopic symptoms through activation of cross-talking signaling cascades. Altered accumulation and phosphorylation of host proteins were identified in early studies (for a review see Navarro et al., 2012a), which more recently have been complemented by genome-wide transcriptome analyses (Qi and Ding, 2003; Wang et al., 2011; Owens et al., 2012; Rizza et al., 2012). Although it remains to be conclusively established whether the genes with altered expression are actually involved in symptom elicitation, these studies have supplied interesting information for further dissecting the molecular interplay between viroids and their hosts.

In the last decade, new hints on possible mechanisms by which non-protein-coding RNAs, like viroids, could modify host gene expression have been derived from dissection of RNA silencing pathways in plants (for reviews see Chen, 2009; Parent et al., 2012). Since early identification in infected tissues of viroid-derived small RNAs of 21–24 nt (vd-sRNAs) with structural features similar to the small interfering RNA (siRNAs) and micro RNAs (miRNAs; Itaya et al., 2001; Papaefthimiou et al., 2001; Martínez de Alba et al., 2002), RNA silencing has been proposed as a regulatory network by which viroids may modify host gene expression eventually resulting in macroscopic symptoms. In particular, it was proposed that, similarly to miRNAs and siRNAs, vd-sRNAs could be incorporated into Argonaute (AGO) complexes and target host mRNAs for cleavage or translation inhibition. The correlation between symptom severity and vd-sRNAs accumulation (Markarian et al., 2004; Wang et al., 2004; Matoušek et al., 2007; Gómez et al., 2008), and the identification of pre-miRNAs or mRNAs potentially targeted by vd-sRNAs (Diermann et al., 2010; Wang et al., 2011), provided circumstantial support for this hypothesis. However, compelling evidence that vd-sRNAs, mimicking miRNAs, indeed target host mRNAs for sequence-specific cleavage has been supplied only recently by dissecting the molecular mechanisms potentially involved in the induction of PC by PLMVd (Navarro et al., 2012b). More explicitly, two PLMVd-sRNAs containing the insertion strictly associated with PC were identified by vd-sRNAs deep sequencing and bioinformatics tools. RNA ligase-mediated rapid amplification of cDNA ends (RACE) has shown that these vd-sRNAs target for cleavage the mRNAs coding for the chloroplastic heat-shock protein 90 (cHSP90) as predicted by RNA silencing (Figure 2A inset). Interestingly, this protein is involved in chloroplast biogenesis and plastid-to-nucleus-signaling, which appear compromised in the albino tissues characteristic of PC (Rodio et al., 2007). In these tissues accumulation of the mRNA encoding for cHSP90 is significantly lowered with respect to the green adjacent tissues. Altogether these data strongly support a role for cHSP90 down-regulation in PC macroscopic symptoms and the closely associated cytopathic effects, although involvement of additional factors cannot be excluded at this stage (Navarro et al., 2012b). In addition, based on the previous observation that rRNA maturation is impaired in altered plastids from albino sectors of leaves expressing PC (Figure 2B; Rodio et al., 2007), it can be speculated that cHSP90, a chaperone, could mediate directly or indirectly this biochemical pathway.

Are the other ultrastructural defects induced by viroids also due to misregulation of specific host genes? Some years ago, the stunting and restricted cell wall expansion induced by PSTVd in tomato was correlated to the down-regulation of an expansin gene *(LeExp2*; Qi and Ding, 2003). More recently, genome-wide analyses have shown important transcriptional changes in response to infections by CEVd in Etrog citron (Rizza et al., 2012) and PSTVd in tomato (Owens et al., 2012). Notwithstanding the different viroid-host combinations and experimental designs of these studies, it is worth noting that overexpression of genes coding for proteins involved in cell wall remodeling (i.e., pectinesterases) and down-regulation of genes involved in chloroplast metabolism and biogenesis, were observed in both cases. Even if it is not known whether modulation of these genes can be directly related to the ultrastructural alterations in cell wall and chloroplast induced by CEVd and PSTVd, these data are intriguing. In addition, the identification of genes consistently overexpressed in viroid-infected hosts points to the existence of mechanisms, apart from RNA silencing mediated by vd-sRNAs, by which viroids could interfere with host gene expression.

## Concluding Remarks

Ultrastructural defects induced by viroids in their hosts were prevalently investigated by biochemical and electron microscopy techniques in the 70s and 80s of the last century. However, the beginning of the 90s witnessed a change of focus and, since then, studies on viroid pathogenesis have been dominated by molecular approaches, leaving almost completely aside cellular and histological studies. Nowadays, current technologies based on genome-wide analyses, including deep sequencing, are being increasingly applied for dissecting viroid-host interactions at the molecular level, and it can be anticipated that they will be more and more used in the future. However, the studies on PC induced by PLMVd (Rodio et al., 2007; Navarro et al., 2012b) exemplify that data on cytopathic effects must be integrated when trying to characterize the biochemical pathways and regulatory networks involved in plant responses to viroid infections. At the same time, new studies on viroid-induced cytopathic alterations appear particularly stimulating if they are included in a wider experimental design aimed at exploring by high-throughput technologies the concurrent modifications in host gene expression. This combined multidisciplinary strategy should be instrumental for generating a broader view of how these fascinating non-coding RNAs manipulate their hosts.

## Conflict of Interest Statement

The authors declare that the research was conducted in the absence of any commercial or financial relationships that could be construed as a potential conflict of interest.
